# Adjusting retinol-binding protein concentrations for inflammation: Biomarkers Reflecting Inflammation and Nutritional Determinants of Anemia (BRINDA) project

**DOI:** 10.3945/ajcn.116.142166

**Published:** 2017-06-14

**Authors:** Leila M Larson, Sorrel ML Namaste, Anne M Williams, Reina Engle-Stone, O Yaw Addo, Parminder S Suchdev, James P Wirth, Victor Temple, Mary Serdula, Christine A Northrop-Clewes

**Affiliations:** 1 Nutrition and Health Sciences Program, Laney Graduate School, and; 2 Rollins School of Public Health, Emory University, Atlanta, GA;; 3 Strengthening Partnerships, Results, and Innovations in Nutrition Globally, Arlington, VA;; 4 Helen Keller International, Washington DC;; 5 Department of Nutrition, University of California, Davis, CA;; 6 Nutrition Branch, CDC, Atlanta, GA;; 7 GroundWork, Fläsch, Switzerland;; 8 School of Medicine and Health Sciences, University of Papua New Guinea, Port Moresby, Papua New Guinea; and; 9 Independent Public Health Nutrition Consultant, Cambridge, United Kingdom

**Keywords:** anemia, inflammation, meta-analysis, nutritional assessment, retinol-binding protein, vitamin A deficiency

## Abstract

**Background:** The accurate estimation of the prevalence of vitamin A deficiency (VAD) is important in planning and implementing interventions. Retinol-binding protein (RBP) is often used in population surveys to measure vitamin A status, but its interpretation is challenging in settings where inflammation is common because RBP concentrations decrease during the acute-phase response.

**Objectives:** We aimed to assess the relation between RBP concentrations and inflammation and malaria in preschool children (PSC) (age range: 6–59 mo) and women of reproductive age (WRA) (age range: 15–49 y) and to investigate adjustment algorithms to account for these effects.

**Design:** Cross-sectional data from 8 surveys for PSC (*n* = 8803) and 4 surveys for WRA (*n* = 4191) from the Biomarkers Reflecting Inflammation and Nutritional Determinants of Anemia (BRINDA) project were analyzed individually and combined with the use of a meta-analysis. Several approaches were explored to adjust RBP concentrations in PSC in inflammation and malaria settings as follows: *1*) the exclusion of subjects with C-reactive protein (CRP) concentrations >5 mg/L or α-1-acid glycoprotein (AGP) concentrations >1 g/L, *2*) the application of arithmetic correction factors, and *3*) the use of a regression correction approach. The impact of adjustment on the estimated prevalence of VAD (defined as <0.7 μmol/L) was examined in PSC.

**Results:** The relation between estimated VAD and CRP and AGP deciles followed a linear pattern in PSC. In women, the correlations between RBP and CRP and AGP were too weak to justify adjustments for inflammation. Depending on the approach used to adjust for inflammation (CRP+AGP), the estimated prevalence of VAD decreased by a median of 11–18 percentage points in PSC compared with unadjusted values. There was no added effect of adjusting for malaria on the estimated VAD after adjusting for CRP and AGP.

**Conclusions:** The use of regression correction (derived from internal data), which accounts for the severity of inflammation, to estimate the prevalence of VAD in PSC in regions with inflammation and malaria is supported by the analysis of the BRINDA data. These findings contribute to the evidence on adjusting for inflammation when estimating VAD with the use of RBP.

## INTRODUCTION

Vitamin A deficiency (VAD) is a widespread public-health problem with the most vulnerable groups being preschool children (PSC), women of reproductive age (WRA), and pregnant women (1). Vitamin A has a role in many biological functions including growth, vision, epithelial differentiation, immune function, and reproduction (2). VAD is the leading cause of preventable blindness in children and night blindness in pregnant women and increases the risks of disease and death from infections especially in PSC (2, 3).

Serum retinol concentrations reflect liver vitamin A stores only when liver stores are severely depleted (< 0.07 μmol/g liver) or extremely high (>1.05 μmol/g liver) (4). However, the population distribution of serum retinol concentrations or its carrier retinol-binding protein (RBP) or the prevalence of individuals with serum retinol or RBP concentrations <0.7 μmol/L can provide important information about the vitamin A status of a population and may reflect the severity of VAD as a public-health problem (5). Studies have shown an ∼1:1 relation between serum retinol and RBP, and therefore, RBP is often substituted as an indicator of vitamin A status (6) with the advantage of RBP being more robust for sample collection and handling processes and less expensive to measure (7).

Inflammation and nutrition are closely related (8) with the result that many nutritional biomarkers, such as serum retinol and RBP, are altered in the presence of inflammation (9). During the inflammatory process, there are rapid and large changes in the plasma concentrations of retinol and RBP, the latter of which is itself a negative acute-phase protein (APP) (10). Two of the most commonly measured APPs to reflect an individual’s inflammatory response in cross-sectional surveys are C-reactive protein (CRP), which rises rapidly and acutely in response to an inflammatory stimulus, and α-1-acid glycoprotein (AGP), which has a slower and longer response (11, 12).

To our knowledge, there are few studies in which biomarkers of vitamin A have been measured before, during, and after physical trauma. Louw et al. (10) studied the “natural history” of blood vitamin concentration status in adults who were undergoing elective surgery and showed that plasma concentrations of retinol and RBP decreased significantly by 48 h and mirrored the changes in CRP. These changes were transient, and serum retinol and RBP concentrations returned to preoperation concentrations 7 d postsurgery (10).

The regions with the highest prevalence of VAD often also have the greatest burden of malaria. This association is important because the effects of malaria on vitamin A status can be categorized by transient reductions from decreased RBP synthesis by the liver, decreased release of the retinol-RBP complex into the circulation, and leakage into the extravascular space because of increased vascular permeability (12–14). Some of these effects may be mediated by the acute-phase response; however, the specific contribution of malaria infection in temporarily altering serum retinol or RBP concentrations beyond the acute-phase response is unclear.

In surveys of RBP, an important question is whether to account for inflammation in estimating the prevalence of VAD and, if so, by what approach. One option is to exclude respondents with elevated APP values; however, the proportion of individuals with elevated APPs can be high in some populations, and thus, the exclusion of these individuals leads to less precision. To address this issue, Thurnham et al. (15) conducted a meta-analysis to generate correction factors (CFs) to adjust individuals’ serum retinol concentrations on the basis of 4 stages of subclinical infection. A more recent approach suggests removing the effect of inflammation on retinol or RBP with the use of a regression approach (16, 17). No consensus has been reached on which approach should be applied to interpret serum retinol or RBP concentrations to account for inflammation in the assessment of vitamin A status in populations. The WHO has no recommendation on how to adjust serum retinol or RBP for inflammation (5).

To better inform nutrition programs and policies, the current study describes different approaches to adjust RBP and estimate the prevalence of VAD in PSC and nonpregnant WRA in settings with a high prevalence of inflammation. In this paper, the following 4 questions are explored: *1*) Is there a need to measure biomarkers of inflammation when RBP is used as an indicator of VAD? *2*) Is there a need to measure >1 inflammation biomarker (CRP and AGP) to adjust RBP? *3*) Is an additional adjustment needed to correct RBP concentrations in the presence of malaria? and *4*) How do the different adjustment approaches for correcting for inflammation and for calculating the estimated prevalence of VAD compare with each other?

## METHODS

We used surveys from the Biomarkers Reflecting Inflammation and Nutritional Determinants of Anemia (BRINDA) project (www.BRINDA-nutrition.org) (17). The BRINDA protocol was reviewed by the institutional review boards of the NIH and was deemed to be non–human subjects research. The methods for identifying data sets, inclusion and exclusion criteria, and data management for the BRINDA project have been described in the methodologic overview in this supplement, which is an open access publication (18). Briefly, the surveys were nationally or regionally representative, and the inclusion criteria were as follows: *1*) surveys were conducted after 2004, *2*) target groups included PSC and/or nonpregnant WRA, and *3*) surveys measured ≥1 marker of iron (ferritin or soluble transferrin receptor) or vitamin A status (RBP or retinol) and ≥1 marker of inflammation (AGP or CRP). Surveys that were included in this analysis were those with measures of RBP and inflammation (CRP and/or AGP). Originally, surveys that measured serum retinol were also included; however, only 4 surveys measured retinol, 2 surveys of which measured only CRP, and the other 2 surveys measured only AGP. Because of the lack of comparability between the surveys that included serum retinol, only surveys that measured RBP are presented here. Of the 16 PSC and 10 WRA BRINDA data sets, data from 8 PSC and 4 WRA data sets were available for analysis for this paper. In all surveys in which WRA data were collected, PSC data were also collected as part of the same surveys. Both CRP and AGP were measured in all data sets, and malaria was measured in 5 PSC and 3 WRA data sets.

### Laboratory analysis

Venous or capillary blood was collected from each respondent, and plasma or serum was stored at −20°C until analysis; one survey used dried blood spots. RBP, CRP, and AGP concentrations were assessed with the use of a sandwich ELISA at the VitMin Laboratory in all data sets (7). Current malaria was assessed with the use of microscopy in Kenya and Côte d’Ivoire (19), and current or recent malaria was assessed with the use of the Paracheck Pf rapid diagnostic test (Orchid Biomedical System) in Liberia and plasma histidine rich protein 2 (Cellabs Pty Ltd.) in Cameroon. Additional information on laboratory methods is further described in the methodologic overview in this supplement (18).

### Case definitions

RBP concentrations <0.7 μmol/L in PSC (5) were used to define VAD, and RBP concentrations <1.05 μmol/L in WRA were used to define vitamin A insufficiency (VAI) (20–24). The use of a concentration cutoff of <0.7 μmol/L in WRA resulted in a prevalence of VAD ≤2% across all surveys. Malaria was defined as either positive or negative. Inflammation was defined as a CRP concentration >5 mg/L and/or AGP concentration >1 g/L (7, 15).

The household socioeconomic status asset score was defined by survey investigators who applied a principal component analysis within each survey to household characteristics and item ownership for comparison of the poorest wealth quintile to the higher wealth quintiles. Maternal education was defined as any school compared with no school.

### Statistical analysis

Descriptive statistics were calculated with the use of SAS 9.4 software (SAS Institute) and cross-checked with SAS or STATA 12.0 software (StataCorp). Correlations between RBP, CRP, and AGP concentrations and malaria were calculated with the use of Kendall’s τ coefficient with the SOMERSD package (25). The Taylor linearization method was used to obtain unbiased estimates that incorporated the sampling weight, strata, and cluster (as applicable) when analyzing individual surveys. First, individual survey analyses, accounting for the complex survey design, were done with the use of the survey package in R 3.2.2 software (R Core Team) (26). Individual survey estimates were combined with the use of a meta-analysis approach with the metafor package in R 3.2.2 software (R Core Team) (27, 28). The heterogeneity of estimates across the surveys was assessed with the use of Cochrane’s heterogeneity test (27, 28).

The prevalences of VAD and VAI were estimated without any adjustments to RBP and were referred to as unadjusted estimates. The following 3 approaches were used to adjust RBP for inflammation and malaria: exclusion, CF, and regression corrections (RC) (18). Because of the weak relation between inflammation and RBP in women, as described in Results, adjustment approaches were not pursued in the WRA data.

### Exclusion approach

The exclusion approach uses the inflammation biomarker information to exclude individuals with elevated CRP and/or AGP (defined as a CRP concentration >5 mg/L and/or AGP concentration >1 g/L) from the analysis; this exclusion results in a smaller sample size. We excluded these individuals and calculated the estimated prevalence of VAD in the remaining individuals.

### CF approach

The CF approach, as proposed by Thurnham et al. (15), uses arithmetic CFs. We calculated CFs by grouping inflammation into 4 groups as follows: *1*) reference (both CRP concentration ≤5 mg/L and AGP concentration ≤1 g/L); *2*) incubation (CRP concentration >5 mg/L and AGP concentration ≤1 g/L); *3*) early convalescence (both CRP concentration >5 mg/L and AGP concentration >1 g/L); and *4*) late convalescence (CRP concentration ≤5 mg/L and AGP concentration >1 g/L). We also calculated CFs by grouping inflammation or malaria into 2 groups in which CRP, AGP, or malaria was used independently. CFs were defined as the ratio of geometric means of the reference group (nonelevated CRP and/or AGP or malaria negative depending on the grouping used) to those of the respective inflammation group or malaria-positive group. CFs were calculated based on internal survey-specific data [termed the Internal Correction Factor (ICF)], a previous meta-analysis using distinct data from the BRINDA data set [termed Thurnham Correction Factor (TCF)] (15), and the meta-analyzed BRINDA data set [termed BRINDA Correction Factor (BCF)]. The CF approach was performed for CRP alone, AGP alone, and both CRP and AGP. CFs were additionally calculated for malaria with the use of the ICF approach only. Although the Thurnham meta-analysis (15) looked specifically at serum retinol, serum retinol and RBP concentrations have consistently been shown to be highly correlated (29). Hence, the TCFs have been used to adjust RBP for inflammation.

### RC approach

The RC approach uses linear regression to adjust RBP by the concentration of CRP and/or AGP on a continuous scale and malaria as a dichotomous variable as defined further in the BRINDA methods article (18). Briefly, the adjusted RBP equation was calculated by subtracting the influence of CRP, AGP, and malaria as follows:


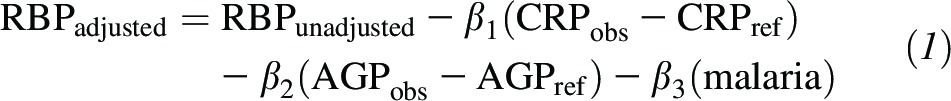


Depending on the available data, CRP, AGP, and/or malaria can be included in the model. β_1_ is the CRP regression coefficient, β_2_ is the AGP regression coefficient, β_3_ is the malaria regression coefficient, obs is the observed value, and ref is the reference value (maximum value of the lowest CRP or AGP decile with the use of combined BRINDA data with nonlogged reference values of CRP in PSC of 0.10 and of AGP in PSC of 0.59). CRP, AGP, and RBP are all ln transformed; CRP and AGP are continuous variables, and malaria is a dichotomous variable. The correction was only applied to individuals with ln CRP greater than ln CRP_ref_ and ln AGP greater than ln AGP_ref_ to avoid overadjustments (18). An illustrative example of the use of the RC approach to adjust RBP for AGP and CRP in PSC in Liberia is provided in **Supplemental Figure 1**.

The first step in the RC approach was to ln transform RBP, CRP, and AGP concentrations to approximate normality on the basis of regression diagnostics. CRP data contained values of zero, and thus, the survey-specific lowest value was used to replace zeros before applying the ln transformation. Second, linear regression coefficients for CRP, AGP, and/or malaria were obtained (bivariate and multiple) with RBP as the outcome. A test of multicollinearity between ln CRP and ln AGP or between malaria and ln CRP and/or ln AGP was assessed on the basis of a test of tolerance (>0.1) and variance inflation factor (<5) to determine whether it was appropriate to include all variables in the model. Third, a ln CRP_ref_ was subtracted from ln-CRP concentrations, and a ln AGP_ref_ was subtracted from ln-AGP concentrations in the regression equation.

The RC approach is presented based on internal survey specific data [termed the Internal Regression Correction (IRC)] and the meta-analyzed BRINDA data [termed BRINDA Regression Correction (BRC)]. The BRC approach entailed replacing the CRP and AGP β coefficients in the adjusted RBP Equation *1* with the meta-analysis β coefficients. The same external reference values for CRP and AGP were used when applying both the IRC and BRC approaches. Each of these RCs was performed for CRP alone, AGP alone, and both CRP and AGP. The IRC was also performed for malaria alone, and malaria plus CRP and AGP. The BRC approach was not applied when including malaria in the model because of the limited number of data sets that measured malaria.

We also tested whether potential confounders significantly influenced the relation between RBP and inflammation. We added sex, age, maternal education, and socioeconomic status to the model, and the CRP and AGP slopes that were adjusted for these confounders were extracted and used in the adjusted RBP equation.

### Comparison of adjustments

Unadjusted and adjusted prevalence estimates of VAD were compared with the use of McNemar’s chi-square test; statistical significance was defined as *P* < 0.05 before applying the Bonferroni corrections to account for multiple comparisons (*P* = 0.05 ÷ *k*, where *k* equals the number of comparisons). To examine whether malaria infection remained associated with VAD after adjustment for CRP and AGP, we stratified the adjusted prevalence of VAD estimates by malaria status and compared the adjusted VAD prevalence with the use of the adjusted Wald test. It was decided a priori that adjustments for CRP and AGP together and CRP, AGP, and malaria combined would be explored irrespective of the significance between malaria and nonmalaria VAD prevalence.

## RESULTS

### Participant characteristics

Our study sample was restricted to participants with no missing values for RBP, CRP, AGP, or malaria (in surveys that measured malaria). The analysis included a total of 8803 PSC and 4191 WRA. Participants who were excluded because of missing RBP, CRP, AGP, or malaria data (6.6%) did not differ from those who were included with regard to sex, age, or socioeconomic status. The PSC data sets had a wide disparity in age ranges as follows: 6–12 mo (*n* = 1 data set), 6–23 mo (*n* = 1 data set), 6–35 mo (*n* = 3 data sets), 12–59 mo (*n* = 1 data set), and 6–59 mo (*n* = 2 data sets) (**Table 1**). The geometric mean of RBP concentrations was lower in children (0.81–1.02 μmol/L) than in women (1.32–1.57 μmol/L) in all countries. Similarly, the prevalence of any inflammation (defined as elevated CRP and/or AGP) was higher in children (26.0–67.5%) than in women (19.7–33.7%) in all countries. The prevalence of malaria also differed across surveys with malaria data (Cameroon, Côte d’Ivoire, Kenya, and Liberia), and ranged from 19.7% to 32.5% in children and from 5.0% to 17.9% in women (Table 1).

**TABLE 1:** Age, inflammation, and malaria in preschool children and women of reproductive age: the BRINDA project^1^

Survey	*n*	Age^2^	CRP concentration >5 mg/L	AGP concentration >1 g/L	CRP concentration >5 mg/L or AGP concentration >1 g/L	Malaria
Preschool children						
Bangladesh	1493	8.3 (6, 11)	14.3 (11.8, 16.7)	33.4 (29.9, 36.9)	35.8 (32.2, 39.5)	—
Cameroon	774	30.8 (12, 59)	37.5 (32.7, 42.3)	39.3 (33.7, 45.0)	48.3 (43.2, 53.5)	25.9 (20.2, 31.5)
Côte d’Ivoire	733	31.7 (6, 59)	40.4 (36.5, 44.3)	64.5 (60.3, 68.6)	67.5 (63.8, 71.3)	27.2 (22.3, 32.0)
Kenya 2007	888	19.9 (6, 35)	27.8 (23.9, 31.7)	64.2 (60.2, 68.2)	66.0 (61.9, 70.1)	19.7 (15.8, 23.6)
Kenya 2010	843	21.4 (6, 35)	34.2 (29.6, 38.7)	60.7 (56.0, 65.4)	61.9 (57.2, 66.6)	32.5 (28.4, 36.6)
Liberia	1434	19.9 (6, 35)	29.5 (26.5, 32.5)	56.2 (52.5, 60.0)	59.1 (55.6, 62.7)	29.4 (26.2, 32.6)
Papua New Guinea	871	37.9 (6, 59)	31.6 (27.2, 36.0)	54.1 (49.4, 58.9)	57.0 (52.6, 61.5)	—
Philippines	1767	15.0 (6, 23)	13.9 (11.6, 16.2)	21.2 (17.7, 24.6)	26.0 (22.4, 29.5)	—
Women of reproductive age						
Cameroon	751	27.2 (15, 49)	17.8 (14.8, 20.7)	7.2 (5.1, 9.3)	19.7 (16.6, 22.9)	15.0 (11.3, 18.6)
Côte d’Ivoire	816	27.6 (15, 49)	19.7 (16.5, 22.8)	26.9 (23.5, 30.4)	33.7 (29.6, 37.9)	5.0 (3.4, 6.5)
Liberia	1875	28.6 (15, 49)	14.3 (12.1, 16.4)	10.4 (8.7, 12.2)	18.5 (16.2, 20.8)	17.9 (15.3, 20.4)
Papua New Guinea	749	33.5 (15, 49)	10.0 (7.5, 12.5)	21.8 (18.1, 25.6)	24.8 (21.0, 28.7)	—

1 Values are percentages (95% CIs) unless otherwise indicated. Elevated CRP and AGP concentrations were defined as >5 mg/L and >1 g/L, respectively. AGP, α-1-acid glycoprotein; BRINDA, Biomarkers Reflecting Inflammation and Nutritional Determinants of Anemia; CRP, C-reactive protein.

2 All values are means (minimum to maximums in parentheses). Age is given in whole months in children and in whole years in women.

### Relation between RBP, inflammation, and malaria

**FIGURE 1:**
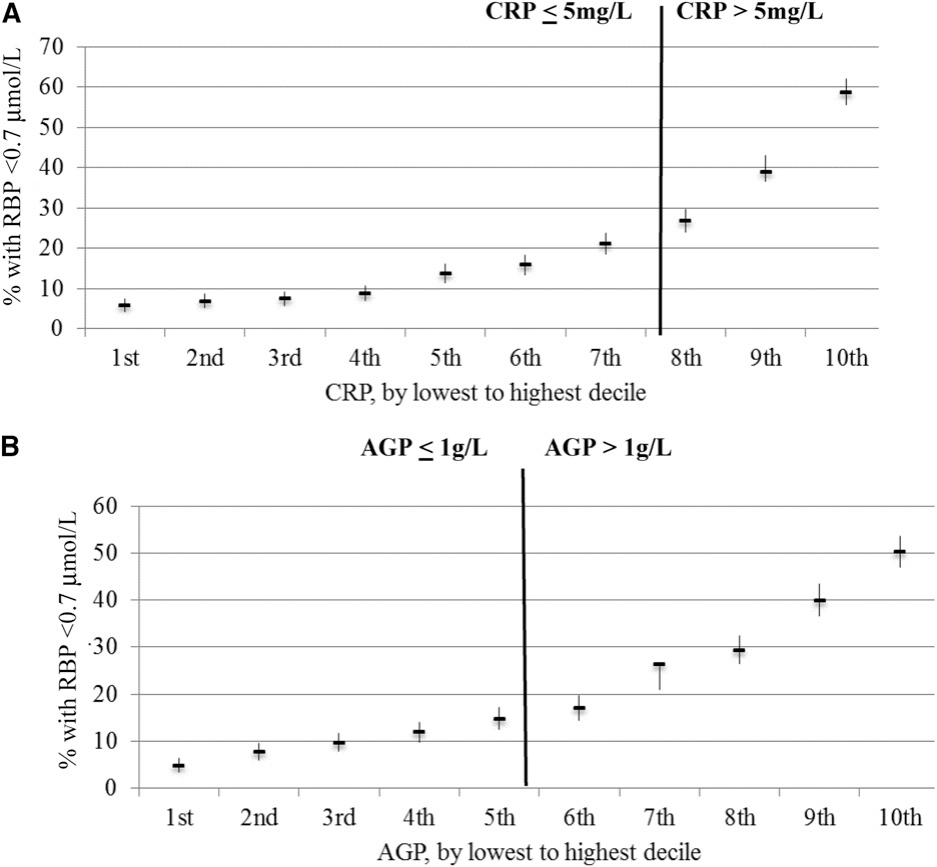
Estimated prevalence [percentage (95% CI)] of vitamin A deficiency in preschool children by CRP (A) and AGP (B) deciles: the Biomarkers Reflecting Inflammation and Nutritional Determinants of Anemia (BRINDA) project. The analysis was restricted to surveys (Bangladesh, Cameroon, Côte d’Ivoire, Kenya 2007, Kenya 2010, Liberia, Philippines, and Papua New Guinea) that measured both CRP and AGP for comparability between CRP and AGP relations with biomarkers (*n* = 8803). Vitamin A deficiency was defined as an RBP concentration <0.70 μmol/L. Bold vertical lines indicate commonly used cutoffs for CRP and AGP. AGP, α-1-acid glycoprotein; CRP, C-reactive protein; RBP, retinol-binding protein.

**FIGURE 2:**
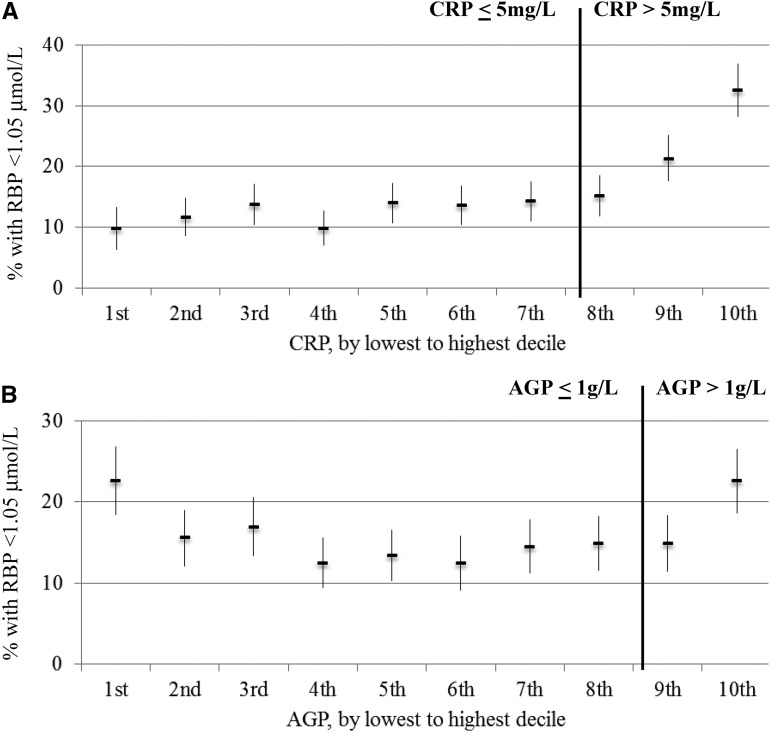
Estimated prevalence [percentage (95% CI)] of vitamin A insufficiency in women of reproductive age by CRP (A) and AGP (B) deciles: the Biomarkers Reflecting Inflammation and Nutritional Determinants of Anemia (BRINDA) project. The analysis was restricted to countries (Cameroon, Côte d’Ivoire, Liberia, and Papua New Guinea) (*n* = 4191).Vitamin A insufficiency was defined as an RBP concentration <1.05 μmol/L. Bold vertical lines indicate commonly used cutoffs for CRP and AGP. AGP, α-1-acid glycoprotein; CRP, C-reactive protein; RBP, retinol-binding protein.

In PSC, Kendall’s τ coefficients between RBP and CRP ranged from −0.26 to −0.37, between RBP and AGP ranged from −0.18 to −0.32, and between CRP and AGP ranged from 0.49 to 0.63 (*P* < 0.05) (**Supplemental Table 1**). In WRA, the correlations of markers of inflammation with RBP were not as strong and were significant (*P* < 0.05) for only CRP in some surveys but not for AGP. Kendall’s τ coefficients between RBP and CRP ranged from −0.02 to −0.13, between RBP and AGP were close to zero, and between CRP and AGP were close to 0.40 (Supplemental Table 1). In PSC and WRA, there was a weak relation between malaria and RBP ranging from −0.10 to −0.19 (*P* < 0.05) in PSC and −0.07 to 0 (*P* > 0.05) in WRA (Supplemental Table 1). The estimated prevalence of VAD in PSC increased with increasing CRP and AGP deciles (**Figure 1**). The relation between the estimated prevalence of VAD and inflammation deciles did not vary according to whether subjects were above or below CRP and AGP cutoffs. This relation was not the case in women (**Figure 2**). Because of the lack of a strong and consistent relation between RBP and CRP and AGP in women, adjustments for inflammation are only presented for children.

### CFs for RBP, inflammation, and malaria

The 4-level ICF values (used in the ICF adjusting for CRP and AGP approach) and 2-level ICF values (used in the ICF adjusting for CRP and ICF adjusting for AGP approaches) were >1 across all categories and surveys in PSC (**Supplemental Table 2**). The CFs were also >1 for malaria across all groups and surveys in PSC (**Supplemental Table 3**). When individuals were stratified by malaria status and by 4-inflammation levels, geometric mean RBP concentrations tended to be lower in the PSC group with malaria and elevated CRP and AGP concentrations, whereas this was not the case for women (**Table 2**). In children, the CFs (BCFs: incubation: 1.22; early convalescence: 1.38; and late convalescence: 1.09) were of similar magnitude to the TCFs that were derived from the previous meta-analysis (15), which used plasma retinol concentrations from 15 studies of apparently healthy individuals (incubation: 1.14; early convalescence: 1.31; and late convalescence: 1.12).

**TABLE 2:** RPB in preschool children and women of reproductive age according to inflammation stage and malaria status: the BRINDA data set^1^

	Malaria-endemic countries			
	Malaria negative		Malaria positive	
Inflammatory stage	*n*	RBP, μmol/L	*n*	RBP, μmol/L
Preschool children^2^				
Reference	1693	0.96 (0.92, 1.00)^3^	201	0.91 (0.87, 0.95)
Incubation	123	0.83 (0.76, 0.91)	37	0.80 (0.68, 0.92)
Early convalescence	669	0.74 (0.70, 0.77)	666	0.65 (0.62, 0.68)
Late convalescence	992	0.90 (0.86, 0.95)	291	0.83 (0.79, 0.86)
Women of reproductive age^4^				
Reference	2401	1.41 (1.32, 1.51)	267	1.33 (1.19, 1.48)
Incubation	230	1.30 (1.20, 1.40)	76	1.13 (1.06, 1.22)
Early convalescence	190	1.19 (1.05, 1.34)	67	1.18 (0.86, 1.61)
Late convalescence	185	1.58 (1.50, 1.67)	26	1.33 (1.07, 1.65)

1 Reference is defined as a CRP concentration ≤5 mg/L and AGP concentration ≤1 g/L; incubation is defined as a CRP concentration >5 mg/L and AGP concentration ≤1 g/L; early convalescence is defined as a CRP concentration >5 mg/L and AGP concentration >1 g/L; and late convalescence is defined as an AGP concentration >1 g/L and CRP concentration ≤5 mg/L. AGP, α-1-acid glycoprotein; BRINDA, Biomarkers Reflecting Inflammation and Nutritional Determinants of Anemia; CRP, C-reactive protein; RBP, retinol-binding protein.

2 Countries in which malaria was measured in children were Cameroon, Côte d’Ivoire, Kenya 2007, Kenya 2010, and Liberia.

3 Geometric mean; 95% CI in parentheses (all such values)

4 Countries in which malaria was measured in women were Cameroon, Côte d’Ivoire, and Liberia.

### RC slopes for RBP, inflammation, and malaria

A multiple linear regression for the internal regression correction adjusting for C-reactive protein and α-1-acid glycoprotein (IRC-CRP+AGP) with ln-RBP as the outcome in PSC resulted in an unstandardized ln-CRP slope ranging from −0.07 (Côte d’Ivoire) to −0.05 (Kenya 2007) and a ln-AGP slope ranging from −0.17 (Kenya 2010) to 0.03 (Papua New Guinea) (**Supplemental Table 4**). The addition of malaria to the multiple regression analysis dampened the ln-CRP and ln-AGP slopes. The bivariate linear regression (for the IRC adjusting for CRP approach and the IRC adjusting for AGP approach) resulted in larger negative slopes for ln-CRP and ln-AGP than in the multiple regression (**Supplemental Table 5**). There was no multicollinearity between ln-CRP and ln-AGP or between ln-CRP, ln-AGP, and malaria, although the larger negative slopes that were seen in the bivariate regression compared with the multiple regression were likely due to ln-CRP and ln-AGP being correlated. Coefficients for the BRC approach are presented in Supplemental Table 4 under All Surveys (BRC adjusting for CRP and AGP) and in Supplemental Table 5 (for BRC adjusting for CRP and BRC adjusting for AGP).

### Unadjusted prevalence of VAD in PSC and VAI in WRA

There was considerable variation in the unadjusted estimated prevalence of VAD in PSC in data sets. Ignoring inflammation resulted in an unadjusted estimated VAD prevalence ranging from 6.9% in the Philippines to 29.5% in Kenya’s 2010 data set (**Table 3**). In WRA, the unadjusted VAI prevalence ranged from 7.6% in Papua New Guinea, to 13.3% in Côte d’Ivoire, to 16.4% in Cameroon, to 20.5% in Liberia.

**TABLE 3:** Estimated prevalence of vitamin A deficiency unadjusted and after excluding those with inflammation in preschool children: the BRINDA data set^1^

	Unadjusted		CRP concentration ≤5 mg/L^2^		AGP concentration ≤1 g/L^2^		CRP concentration ≤5 mg/L or AGP concentration ≤1 g/L^2^	
Survey	*n*	% (95% CI)	*n*	% (95% CI)	*n*	% (95% CI)	*n*	% (95% CI)
Bangladesh	1493	17.0 (14.4, 19.7)	1280	12.6 (10.1, 15.1)	994	10.8 (7.7, 13.9)	958	9.6 (6.8, 12.4)
Cameroon	774	28.5 (24.3, 32.7)	493	10.8 (14.3, 22.3)	483	19.2 (14.9, 23.4)	411	16.6 (12.0, 21.2)
Côte d’Ivoire	733	24.0 (20.3, 27.8)	429	13.1 (10.1, 16.0)	250	10.4 (6.6, 14.1)	227	8.7 (5.3, 12.0)
Kenya 2007	888	23.0 (19.1, 26.8)	641	12.8 (9.9, 15.7)	318	7.5 (4.4, 10.7)	302	6.3 (3.3, 9.3)
Kenya 2010	843	29.5 (25.7, 33.4)	555	15.7 (12.6, 18.7)	331	12.1 (8.3, 15.8)	321	11.5 (7.9, 15.1)
Liberia	1434	24.7 (21.2, 28.1)	1059	16.5 (13.4, 19.7)	672	12.3 (9.0, 15.5)	633	11.6 (8.4, 14.8)
Papua New Guinea	871	25.0 (20.8, 29.3)	588	15.1 (11.7, 18.5)	395	14.3 (10.4, 18.2)	369	12.9 (8.9, 16.9)
Philippines	1767	6.9 (5.1, 8.7)	1500	2.9 (1.8, 4.0)	1345	2.1 (1.3, 3.0)	1274	1.4 (0.8, 1.9)

1 Vitamin A deficiency was defined as a retinol-binding protein concentration <0.70 μmol/L. AGP, α-1-acid glycoprotein; BRINDA, Biomarkers Reflecting Inflammation and Nutritional Determinants of Anemia; CRP, C-reactive protein.

2 Vitamin A deficiency in the subset of samples with nonelevated inflammatory biomarkers.

### Estimated prevalence of VAD in PSC excluding individuals with inflammation

In PSC, the exclusion of individuals with elevated CRP or AGP resulted in a sample-size loss of 28–69% (Table 3). In PSC, the exclusion of individuals with elevated CRP compared with the inclusion of all children from the survey resulted in a decrease in the estimated prevalence of VAD, the largest of which was observed in Cameroon (28.5% compared with 10.8%) (Table 3). This decrease in estimated VAD was also seen when children with elevated AGP were excluded compared with the inclusion of all children; the largest decrease was observed in Kenya’s 2010 data set (29.5% compared with 12.1%, respectively) (Table 3). When children with elevated CRP and/or AGP were excluded compared with the inclusion of all children, the largest decrease was observed in Kenya’s 2007 data set (23.0% compared with 6.3%, respectively) (Table 3).

### Effect of adjusting RBP for inflammation with the use of CRP, AGP, or both CRP and AGP by the CF and RC approaches

**FIGURE 3:**
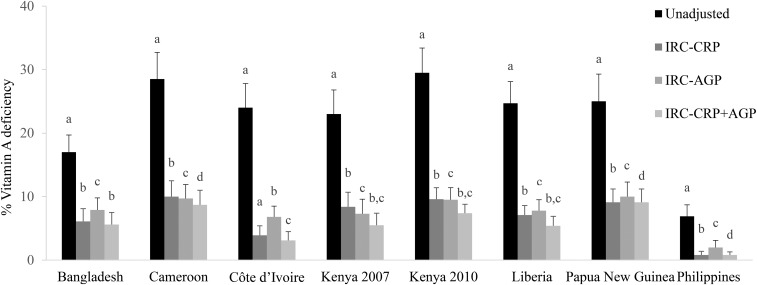
Estimated prevalence [percentage (95% CI)] of vitamin A deficiency on the basis of IRC-CRP, IRC-AGP, or IRC-CRP+AGP in preschool children: the Biomarkers Reflecting Inflammation and Nutritional Determinants of Anemia (BRINDA) project. Vitamin A deficiency prevalence was defined as retinol binding protein <0.7 mmol/L. Bars without a common lowercase letter within a given survey differ, *P* < 0.05 (adjusted by using Bonferroni correction). Internal regression correction reference values were as follows: C-reactive protein: −2.26 ln(mg/L) [QE (df = 10) = 439.90, *P* < 0.0001]; α-1-acid glycoprotein: −0.52 ln(g/L) [QE (df = 10) = 584.5546, *P* < 0.0001]. IRC-AGP, internal regression correction adjusting for α-1-acid glycoprotein; IRC-CRP, internal regression correction adjusting for C-reactive protein; IRC-CRP+AGP, internal regression correction adjusting for C-reactive protein and α-1-acid glycoprotein; QE, QE test of residual heterogeneity.

In PSC, adjustment for CRP alone resulted in the smallest change in VAD in nearly all data sets, which was followed by the change with AGP adjustments and by the change with adjustments for both CRP and AGP compared with unadjusted values. This same pattern was shown across adjustment methods (Supplemental Table 2, **Supplemental Tables 6**–**9**) except with the IRC approach whereby adjusting for CRP alone resulted in a larger decrease in estimated VAD than did adjusting for AGP alone. For instance, with the use of the IRC approach, adjusting for only CRP compared with adjusting for only AGP resulted in a median decrease in the estimated VAD prevalence of 0.8 percentage points (pps). With the use of the IRC approach to adjust for both CRP and AGP compared with adjusting for only CRP resulted in a median decrease in the estimated VAD prevalence of 1.1 pps (**Figure 3**, Supplemental Table 8).

### Comparison of estimated prevalence of VAD in children with the use of different approaches to adjust RBP for inflammation

**FIGURE 4:**
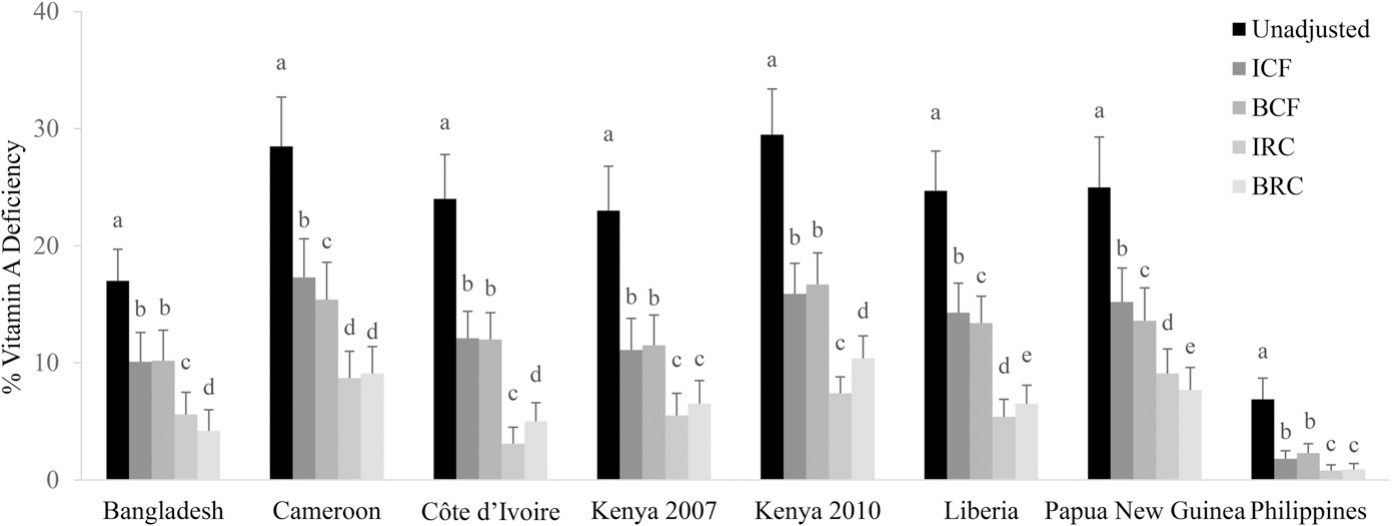
Estimated prevalence [percentage (95% CI)] of vitamin A deficiency with the use of different inflammation-adjustment approaches adjusting for CRP and AGP in preschool children: the Biomarkers Reflecting Inflammation and Nutrition Determinants of Anemia (BRINDA) project. Vitamin A deficiency was defined as retinol binding protein <0.7 μmol/L. Bars without a common lowercase letter within a given survey differ, *P* < 0.05 (adjusted by using Bonferroni correction). BCFs were as follows: incubation phase: 1.22 (95% CI: 1.16, 1.28); early convalescence phase: 1.38 (95% CI: 1.31, 1.44); and late convalescence phase: 1.09 (95% CI: 1.06, 1.11) [QE (df = 28) = 261.6964, *P* < 0.0001). IRC and BRC reference values were as follows: ln CRP = −2.26 ln(mg/L) [QE (df = 10) = 439.90, *P* < 0.0001]; ln AGP = −0.52 ln(g/L) [QE (df = 10) = 584.5546, *P* < 0.0001]. BRC coefficients were as follows: ln CRP = −0.06; and ln AGP = −0.09 [QE (df = 21) = 240.4563, *P* < 0.0001]. AGP, α-1-acid glycoprotein; BCF, Biomarkers Reflecting Inflammation and Nutritional Determinants of Anemia correction factor; BRC, Biomarkers Reflecting Inflammation and Nutritional Determinants of Anemia regression correction; CRP, C-reactive protein; ICF, internal correction factor; IRC, internal regression correction; QE, QE test of residual heterogeneity.

The prevalence of VAD decreased compared with the unadjusted prevalence in PSC when adjusted for CRP and AGP in all surveys regardless of the adjustment approach (**Figures 4** and , Table 3, Supplemental Tables 2, 6–9). The exclusion of individuals with elevated CRP and AGP resulted in a sample size loss of 28–69% in PSC and, with the use of this method, resulted in an estimated prevalence of VAD that was 12.6 absolute median pps (range: 5.5–18.0 pps) lower than the unadjusted prevalence (Table 3).

The use of ICFs resulted in a median decrease of 10.8 pps in the estimated prevalence of VAD (ranging from 5.1 pps in the Philippines to 13.6 pps in Kenya 2010) (Supplemental Table 2, Figure 4). The use of the TCF approach resulted in a lower estimated prevalence of VAD from unadjusted prevalence values by 4.0 pps in the Philippines where unadjusted VAD was lowest to 12.2 pps in Kenya 2010 where unadjusted VAD was the highest (median decrease: 11.0 pps) (Supplemental Table 6). The use of the BCF approach resulted in a median decrease in the prevalence of VAD of 11.5 pps (range: 4.6–13.1 pps) from unadjusted values (Figure 4, Supplemental Table 7). The use of the IRC approach to adjust RBP concentrations in children decreased the VAD prevalence by a median of 18.4 pps (range: 6.1–22.1 pps), and the use of the BRC approach decreased the prevalence by a median of 17.8 pps (range: 6.0–19.4 pps), compared with unadjusted prevalence values (Figure 4, Supplemental Tables 8, 9).

### Adjusted prevalence of VAD in children with the use of malaria in addition to CRP, AGP, to adjust RBP for inflammation and malaria

**FIGURE 5:**
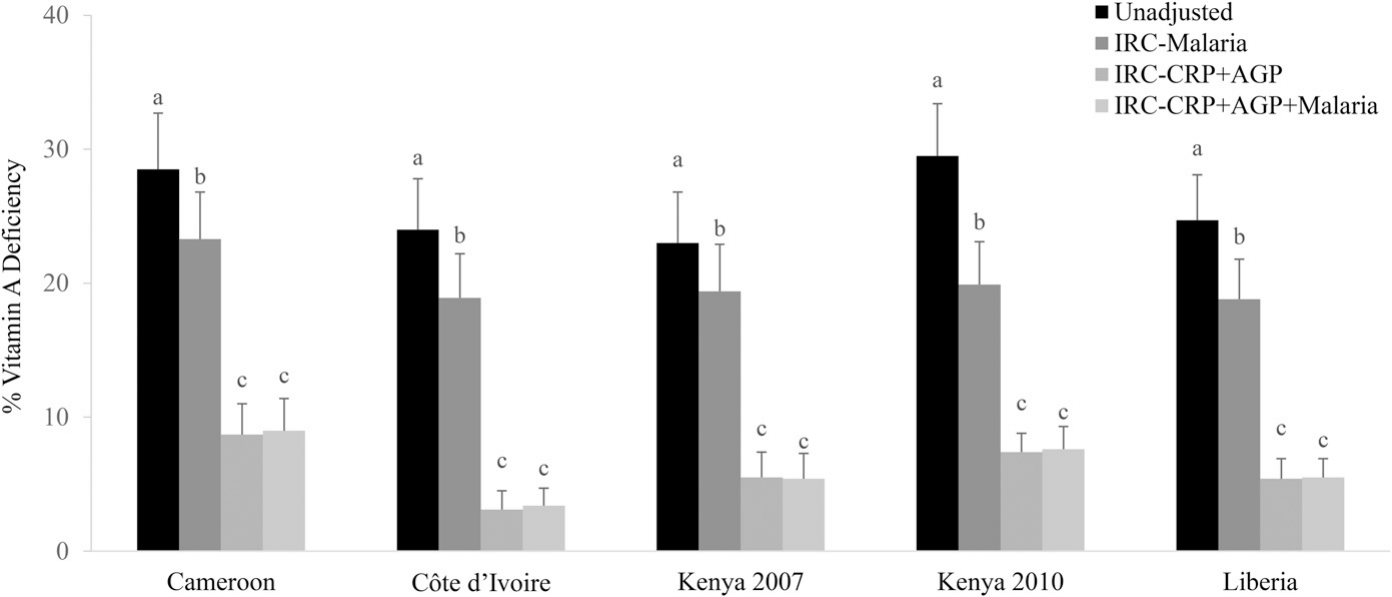
Estimated prevalence [percentage (95% CI)] of vitamin A deficiency in preschool children with the use of different malaria-adjustment approaches. Vitamin A deficiency was defined as retinol-binding protein <0.7 μmol/L. Bars without a common lowercase letter within a given survey differ, *P* < 0.05 (adjusted by using Bonferroni correction). Internal regression correction reference values were as follows: ln C-reactive protein = −2.26 ln(mg/L) [QE (df = 10) = 439.90, *P* < 0.0001]; and ln α-1-acid glycoprotein = −0.52 ln(g/L) [QE (df = 10) = 584.5546, *P* < 0.0001]. IRC-CRP+AGP, internal regression correction adjusting for C-reactive protein and α-1-acid glycoprotein; IRC-CRP+AGP+malaria, internal regression correction adjusting for C-reactive protein, α-1-acid glycoprotein, and malaria; IRC-malaria, internal regression correction adjusting for malaria; QE, QE test of residual heterogeneity.

Adjustment for malaria alone with the use of the ICF approach decreased the estimated prevalence of VAD by an absolute median of 5.2 pps (range: 3.6–9.6 pps) in PSC (Supplemental Table 3). We also examined the effect of malaria in addition to inflammation on RBP concentrations by adjusting RBP concentrations with the use of the regression approach and stratifying the adjusted prevalence of VAD by malaria status. In children, after adjusting RBP concentrations with the use of the IRC approach, the prevalence of VAD significantly differed (*P* < 0.05) in children with and without malaria in 4 of 5 country data sets with malaria data (Côte d’Ivoire, Kenya 2010, Kenya 2007, and Liberia). In these data sets, the difference in the adjusted prevalence of VAD (with the use of IRC) in children with and without malaria ranged from 2% (Liberia) to 7% (Kenya 2010). However, adjusting for inflammation with the use of IRC accounting for both CRP and AGP resulted in similar point estimates of estimated VAD as when adjusting for malaria and inflammation both (Supplemental Table 3, Figure 5).

### Estimated prevalence of VAD with the use of the IRC approach to adjust RBP for inflammation taking into account confounders

There was no change in the prevalence of VAD adjusting for inflammation with the use of the IRC approach in PSC when controlling for socioeconomic status, maternal education, age, and sex (maternal education data were unavailable in Bangladesh, Liberia, and Papua New Guinea; socioeconomic status data were unavailable in Bangladesh) than when not controlling for confounders (data not shown). The absolute median difference of adjusted VAD controlling for confounders compared with not controlling for confounders using IRC adjusting for CRP ranged from −0.3 to 0 pps, using IRC adjusting for AGP ranged from −0.1 to 0.4 pps, and using IRC-CRP+AGP ranged from −0.2 to 0.2 pps.

## DISCUSSION

The findings from this large, multicountry analysis show that RBP concentrations were lower in individuals with elevated CRP and AGP concentrations and/or malaria than in individuals with low inflammation. The adjustment of RBP concentrations with the use of any of the statistical approaches described decreased the estimated prevalence of VAD compared with unadjusted values in children. This decrease was most striking with the use of the RC approach. The adjustments estimated VAD by mathematically removing or reducing the effect of elevated CRP and AGP in population surveys of PSC, which is important for decisions regarding nutrition interventions, programs, and policies.

The first question we explored with this analysis was whether there is a need to measure biomarkers of inflammation when measuring RBP. We showed a strong correlation of both CRP and AGP with RBP in all countries in children, which was expected because of the biological response of the body to inflammation. During the acute-phase response, RBP concentrations may be temporarily reduced because of decreased RBP synthesis, decreased release of the retinol-RBP complex into the circulation, and leakage of RBP into the extravascular space (13). Therefore, during an inflammatory episode, the concentration of RBP is not reflective of the individual’s usual RBP concentration. Because of the effect of the acute-phase response on RBP concentrations, the estimation of VAD prevalence in child populations with high levels of inflammation is likely to be overestimated if no adjustment is made for APPs. However, we showed weak correlations between CRP and AGP with RBP in all surveys with data for women. In addition, the relations between VAI prevalence and CRP decile and VAI prevalence and AGP decile were not linear and did not support the use of the regression method to adjust for inflammation when measuring RBP in women. The reasons for this weak relation and the different patterns in women and children are not well understood but may include the lower prevalence of both inflammation and VAD in women, a more developed immune system in women that allows them to combat infection more efficiently, the potential role of other effect modifiers such as obesity, and the smaller number of surveys. Other authors (15) have suggested applying the same CFs to children and women to adjust serum retinol for inflammation. However, our results suggest that the relations between RBP and CRP and AGP may be different in women than in children. We require additional analyses to explore the potential etiology and whether and how to adjust the results to account for inflammation in populations of women.

Second, we examined the need to measure >1 inflammation biomarker (CRP and/or AGP) to adjust RBP for inflammation in children. The substantial changes in estimates of VAD prevalence from adjustment with the use of the CF and RC approaches than with no adjustment highlight the importance of measuring inflammation. In most cases, adjusting for CRP and AGP together resulted in the largest reduction in the prevalence of VAD in children. Both CRP and AGP are important in explaining the variability in RBP, and their correlation with RBP was similar across countries. CRP has been widely measured and should continue to be measured in conjunction with AGP to better understand their relation with RBP. It would be important to also further investigate the relation between RBP and other inflammatory biomarkers in other settings.

Third, we examined the importance of additional adjustment to correct RBP concentrations in the presence of malaria. Our results show that RBP was associated with malaria independently from CRP and AGP, which was consistent with a recent study that also examined this relation (30). This association could reflect an added influence of malaria-related inflammation on temporary reductions in RBP concentrations that is not captured by CRP and AGP (31). However, the adjustment of RBP for malaria, in addition to CRP and AGP, with the use of the RC approach results in minimal changes in the estimated prevalence of VAD in children compared with values when adjusting for CRP and AGP only. Therefore, there seems to be little added benefit of further adjusting for malaria status to correct RBP concentrations.

Our last question compared adjustment approaches that are used to calculate the prevalence of VAD in the presence of inflammation. In our analysis, the reduction in the estimated prevalence of VAD after adjustment with the use of the RC approach was strong in PSC; in the majority of surveys, the prevalence of VAD dropped by 10–20 pps after adjustment with the use of the IRC adjusting for CRP and AGP approach compared with unadjusted values. Wessells et al. (30) showed a similar drop in VAD prevalence of 11 pps in children 6–23 mo of age in Burkina Faso after adjusting for inflammation with the use of the TCFs adjusting for CRP and AGP approach.

The categorical CFs adjust RBP concentrations on the basis of 4 stages of inflammation that are defined by cutoffs of CRP concentrations >5 mg/L and AGP concentrations >1 g/L. ICFs depend on the inflammation levels and distribution of the population being measured. As such, their precision depends on the number of individuals who fall into each of the 4 stages of inflammation; if this number is too small, the ICFs may not adjust for inflammation correctly. Further, RBP concentrations in children showed a decreasing trend, and VAD prevalence showed an increasing trend, with increasing CRP and AGP concentrations on a continuous scale irrespective of the predefined cutoffs (16–18). Specifically, even in children with CRP and AGP concentrations that were less than the common concentration cutoffs of 5 mg/L and 1 g/L, respectively, CRP and AGP were associated negatively with RBP concentrations. Furthermore, the cutoff of a CRP concentration >5 mg/L was defined at a time when the laboratory methods could not accurately measure lower concentrations (32), and the cutoff for AGP was arbitrarily defined. These associations and the limitations that are inherent in cutoffs provide justification that the RC approach may offer some advantage and precision, by adjusting RBP in a continuous manner that better reflects the association between RBP and APPs over the entire range of APP concentrations.

The severity of inflammation in children seemed to alter RBP concentrations. In PSC, there was consistency in the strength of the linear relation (i.e., slopes with the use of the IRC approach) between RBP and CRP and AGP between countries. The heterogeneity statistic was significant for slopes between countries for PSC, and therefore, the application of an external slope (BRC approach) is not suggested at this time. However, because of the results in PSC, the BRC adjusting for CRP and AGP approach should be further explored in other countries, and if findings are similar to those presented here, the approach could be considered for use across populations. Further investigation is needed to examine potential modifiers of the relation between RBP and CRP and AGP from other causes of inflammation such as tissue injury, intestinal parasites, respiratory infections, aflatoxin, and HIV. RBP may also be altered in the presence of other conditions such as obesity (33) and by age, which should be considered in future investigations.

Although liver vitamin A stores most accurately depict an individual’s vitamin A status (4), serum retinol is the recommended biomarker for determining population-level VAD (20). However, the high costs of the measurements of retinol with the use of HPLC and stability issues make retinol more difficult to use in large population studies than RBP (7). Therefore, RBP is often measured in nutritional surveys and is used to estimate the vitamin A status of a population. To our knowledge, there is currently no consensus on RBP cutoffs to indicate VAD; thus, the VAD-prevalence values that we presented should be interpreted with caution. Our study assumed a molar ratio of retinol:RBP of 1:1 (5, 29); however, different ratios of RBP:retinol have been proposed (23, 34), and some research has suggested that the ratio of RBP and retinol may change during the response to inflammation (35) as the ratio of holo-RBP to apo-RBP changes. When different cutoffs for RBP are used, the regression approach still results in the largest adjustment of the estimated prevalence of VAD. For example, when the Engle-Stone et al. (23) concentration cutoff of 0.83 μmol/L for RBP was used, we observed a median decrease of 25.8 pps (range: 14.0–31.1 pps) with a higher prevalence of estimated VAD overall.

A limitation of the analysis is the nature of cross-sectional data, which does not provide evidence for assessing a causal effect of the inflammatory response on RBP concentrations. The role of vitamin A on the immune response has been well documented (36) and is indicative of the importance of the reverse relation or the effect of VAD on infection. In some cases, the adjustment approaches we present could lead to overcorrection and an underestimation of VAD in certain communities. However, because of the biological plausibility and the large drops we saw in the VAD prevalence after adjustment, we expect the effect of the acute-phase response on RBP to be crucial and warrant the proposed adjustments. Ideally, the use of longitudinal data to examine more deeply the relation between RBP and the acute-phase response could address whether the relation between RBP and biomarkers of inflammation is different between individuals with different vitamin A–status pre-inflammation as well as different causes of inflammation. Although these statistical adjustments are used to create estimates of the prevalence of VAD in population-based studies of children, they do not represent the exact biological influence of inflammation on RBP. The adjustments depicted in this analysis are meant for population-based estimates and are not meant for clinical settings or for the estimation of the vitamin A status of individual children. The current results that were derived from adjustments with CFs and the RC were based on the country data that were available and, therefore, are representative only of the countries presented here. However, because of the similarity in the findings for the countries included and the biological basis for these adjustments, we expect that the same approaches are appropriate for other populations of children with high levels of inflammation.

Nonetheless, the findings of this study highlight the importance of inflammation in interpreting RBP concentrations in populations of children in settings with inflammation. The RC approach allows for a continuous adjustment of RBP by concentrations of CRP and AGP, and this approach appears to yield findings that are sufficiently different from the CF approaches to produce different conclusions on a population’s vitamin A status. These 2 points, and the heterogeneity in BRINDA regression coefficients, support the use of the IRC accounting for both CRP and AGP approach to adjust RBP concentrations and to estimate an adjusted prevalence of VAD in populations of PSC with inflammation (option A). However, the IRC approach requires statistical expertise until there has been a development of a ready-to-use macro. In cases where no such expertise is available, the ICF approach could be used as an alternative approach in children (option B). The ICF approach accounts for population-specific inflammation profiles of which the BCF approach does not, but in case of surveys with a small sample size, the BCF approach can be used. We suggest the use of the newly derived BCFs that are presented in Supplemental Table 7 because they were designed for PSC specifically unlike those shown in the Thurnham et al. (15) meta-analysis, which combined data from pregnant and nonpregnant women, children, neonates, and HIV-positive adults. Any follow-up surveys should use the same approach to monitor trends and maintain comparability.

In conclusion, in settings where inflammation or malaria are present, the burden of VAD may be overestimated. This overestimation is particularly critical in contexts in which vitamin A intervention programs are in place because the expected effects of these programs on vitamin A–status indicators may not be observed if inflammation is ignored; concerns have been raised regarding the potential of excessive vitamin A exposure if vitamin A intervention programs are delivered to individuals with adequate status (37). When data are available and appropriate, a reanalysis of past vitamin A interventions to include the adjustment approach recommended here could yield new insights; relative effects may be larger or smaller from those that were previously shown. The WHO cutoffs to define the degree to which VAD is a public health problem in a population have been prepared on the basis of VAD-prevalence estimates that were unadjusted for inflammation (5); therefore, the cutoffs may not be appropriate compared with adjusted values. A revised recommendation is an issue for the WHO to consider in the future. Thus, the adjusted prevalence estimates presented here cannot be compared with previous estimates that do not account for inflammation (38). This study is meant as a step forward in comparing different approaches to accurately and feasibly assess the prevalence of VAD in populations for CRP and AGP and, when appropriate, malaria.
